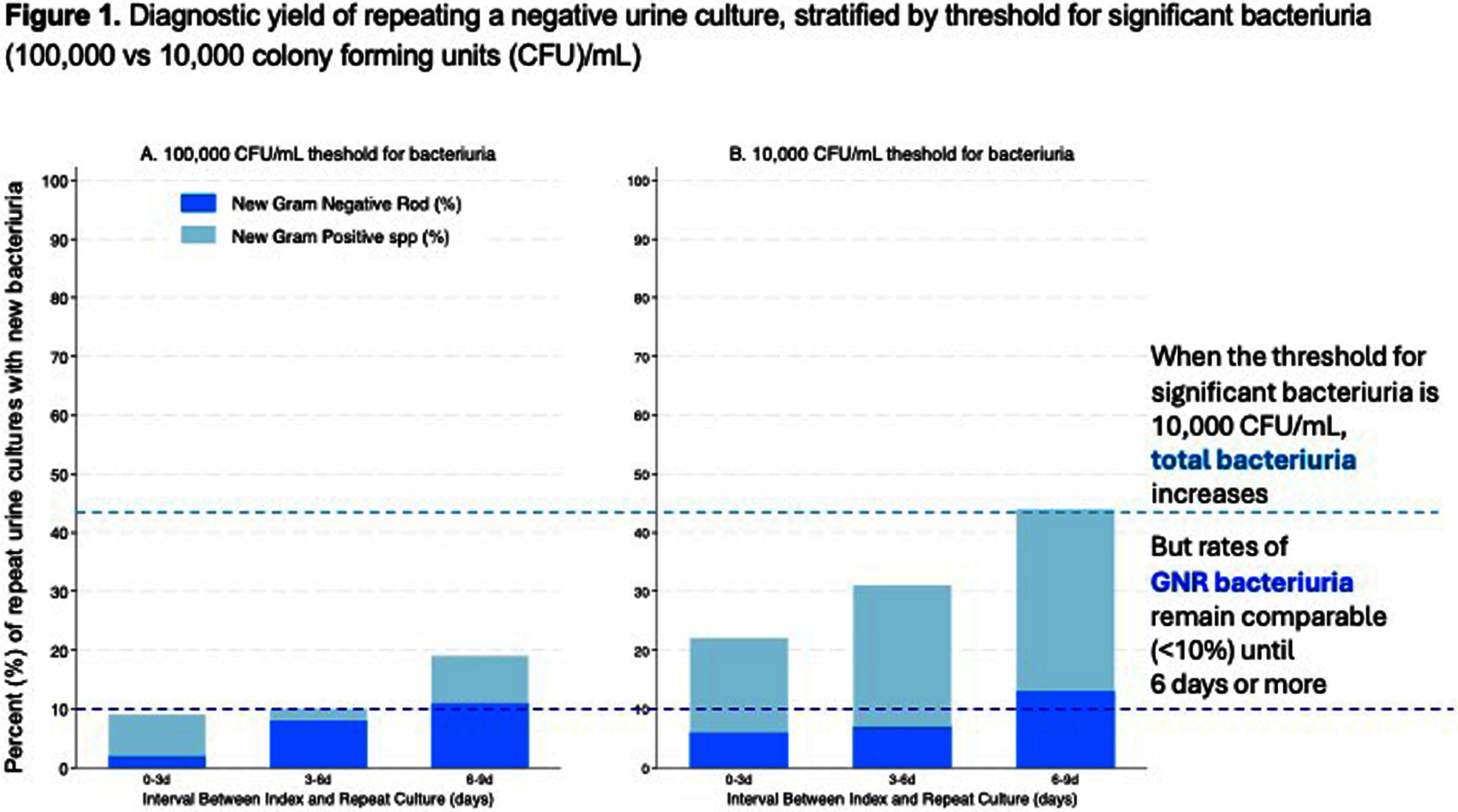# Low Diagnostic Yield of Repeating a Urine Culture in the Inpatient Setting

**DOI:** 10.1017/ash.2025.311

**Published:** 2025-09-24

**Authors:** Eugenia Miranti, Mindy Sampson, John Shepard, Guillermo Rodriguez Nava, Karen McIntyre, Erika Paola Viana Cardenas, Barbara Trautner, Jorge Salinas

**Affiliations:** 1 Stanford Health Care; 2 Stanford University; 3 Stanford University School of Medicine; 4 Baylor College of Medicine

## Abstract

**Background:** Duplicative laboratory testing is prevalent in health care. Prior research surrounding repeat urine cultures showed that when a negative index culture is repeated within 48 hours, less than 5% of repeat urine cultures show a new bacteriuria. We evaluated the diagnostic yield of repeating urine cultures at longer time intervals, and of repeating a positive urine culture. **Methods:** We conducted a retrospective study of adult inpatients at Stanford Healthcare who had more than one urine culture collected during hospitalization between January 2023 and February 2024. We included urine cultures that were collected with or without urinary catheters; nephrostomy tubes were excluded. Urine cultures were classified as index or repeat. We analyzed the diagnostic yield of the repeat urine culture, defined as the percent of repeat urine cultures that detected a new bacteriuria not detected in the index culture. Bacteriuria was defined as growth of a bacterial species in quantities >100,000 CFU/mL. A negative urine culture was defined as one that did not have bacteriuria meeting this threshold. Sensitivity analyses used a threshold of 10,000 CFU/mL as the threshold for significant bacteriuria. **Results:** Overall, 6,955 urine cultures were performed from 6,058 patients. Of these, 864 (12%) urine cultures were repeats. Of the 864 index cultures, 75% were negative. The median time to repeat urine culture was 4 days. When negative index cultures were repeated at 0-3 days, the diagnostic yield for detecting a new bacteriuria was only 9%. Diagnostic yield at 3-6 days was 10%, not significantly higher compared to 0-3 days (p=0.620). Diagnostic yield at 6-9 days was 19%; this increase was significant compared to the 0-3 days group (p=0.014). When positive index cultures were repeated at 0-3 days, the diagnostic yield for detecting a new bacteriuria was only 8%. Diagnostic yield at 3-6 days was also 8%. Yield increased significantly to 15% at 6-9 days from index culture (p=0.013). When the threshold for significant bacteriuria was adjusted to 10,000 CFU/mL, more bacteriuria was detected overall, but primarily of gram-positive organisms. Whether the threshold for significant bacteriuria was 100,000 CFU/mL or 10,000 CFU/mL, the rate of detection of new gram-negative bacteriuria was similar, and remained less than 10% until 6-9 days from index culture (Figure 1). **Conclusions:** Among inpatients, most urine cultures repeated at less than 6 days provide redundant information. This unnecessary retesting offers an opportunity for diagnostic stewardship.

**Figure:**